# Supplementary material for: The relationship between work disability and subsequent suicide or self-harm: A scoping review
Source: PLOS Glob Public Health. 2022 Dec 7;2(12):e0000922. doi: 10.1371/journal.pgph.0000922 (PMC10021753; doi:10.1371/journal.pgph.0000922)
Supplement: S1 Text — (PDF) [file pgph.0000922.s001.pdf]

Work Disability, Suicide and Self-Harm: A Scoping Review Protocol

Authors

Alex Collie[1] and Shannon E Gray[1]

1. School of Public Health and Preventive Medicine, Monash University, Melbourne VIC 3004 Australia.

Abstract

Objective: The objective of this scoping review is to assess and summarise the research literature regarding the relationship between work disability and subsequent suicide or deliberate self-harm.

Introduction: Work disability occurs when a health condition limits the ability of a worker to participate in paid employment. Work disability is common, affecting a substantial proportion of the labour force annually. Several lines of evidence from multiple disciplinary areas suggest that people with work disability are at increased risk of suicide and self-harm. Some of the risk factors for suicide and self-harm are modifiable. There is a need for an exploratory study to examine the relationship between work disability and subsequent suicide and self-harm, and to understand the influence of risk and protective factors.

Inclusion criteria: This review will consider published, peer reviewed studies of people aged 16 years or older with an episode of work disability resulting in absence from work and receipt of financial support. Qualitative, quantitative, and mixed methods studies will be searched.

Methods: Databases to be searched from inception to the present include MEDLINE and Scopus. Studies in English published since the year 2000 will be included. Two independent screeners will screen titles and abstracts as well as full texts of relevant sources. Data will be extracted by a single independent reviewer. Data will be presented in tabular and graphical form accompanied by a narrative summary that aligns with the objective of this scoping review.

Introduction

Work disability occurs when a health condition limits the ability of a worker to participate in paid employment. Common diseases and illnesses of working age are the major causes of work disability, and include conditions with high prevalence such as low back pain, depression, anxiety and traumatic injury. Work disability is common. For every 1000 working age Australians, an estimated 49.4 received income support for a period of work disability from a government or private sector (i.e., not their employer) benefit scheme during the 2015/16 year (1). Several lines of evidence suggest that people with work disability are at increased risk of suicide and self-harm.

First, work disability by definition results in a period of detachment from the workplace, and in some cases may result in unemployment. Unemployment both impairs mental health (2) and is associated with an increased risk of suicide, with the greatest risk within the first five years of unemployment (3).

Second, several studies show that a substantial proportion of people with work disability have mental health conditions, including people in whom the episode of work disability is linked to a physical injury/condition. For example, one Australian study demonstrated that 38% of people with workers' compensation claims for musculoskeletal disorders reported moderate to severe psychological distress (4), while mental health conditions are now the most common medical condition among people receiving social assistance disability benefits (5).

Third, people with work disability are often involved in administrative benefit systems such as workers' compensation or social assistance schemes. A large proportion of people experience the bureaucratic processes involved in eligibility determination and benefit delivery to be stressful (6). Qualitative studies demonstrate that in some people, these administrative processes may lead to long-term mental health problems including suicidal ideation, and reduced quality of life (7, 8).

Fourth, work disability is associated with financial distress. People receiving work disability benefits from workers' compensation and social assistance schemes report high levels of financial distress (9). Financial hardship is frequently cited as a risk factor for suicidal behaviour.

Fifth, long periods of work disability are associated with changes in social support networks, including increasing the burden on caregivers and changing the nature of intimate relationships (10). Social support is a protective factor that reduces the risk of suicide death in adults (11) that may be adversely impacted during periods of work disability.

Finally, many people with work disability and concurrent mental health problems do not receive appropriate mental health treatment, and thus the risk of self-harm or future suicide is more likely to go unrecognised. One study in Australian Disability Support Pension recipients identified that although 69% reported a diagnosed mental health problem, only 16% reported receiving specialist mental health care (12).

Suicide and self-harm are important public health and social problems, and warrant close attention. People with work disability have multiple risk factors for suicide and suicidal behaviour, some of which are modifiable.

Internationally, there are many different ways in which governments and employers seek to support and rehabilitate people experiencing work disability. These include via social security, sickness absence, workers' compensation, life insurance, disability insurance and employment injury insurance systems. These systems can vary substantially with respect to the eligibility, coverage of the labour force, benefit provision and the nature and timing of service provision. People with work disability may also transition between these systems as an episode of work disability extends, or due to changes in their personal or employment circumstances (1). Work disability can also arise from any number of health conditions, injuries or disabilities and can be affected by multiple features of the disabled person and their environment. These determinants of work disability can also interact in complex ways to influence recovery and health (13) including mental health. In summary, work disability is a complex concept. There is a need for an exploratory study to examine the relationship between work disability and subsequent suicide and self-harm.

A preliminary search of MEDLINE and the Cochrane Database of Systematic Reviews was conducted and no current or underway systematic reviews or scoping reviews on the topic were identified.

The objective of this scoping review is to assess and summarise the research literature regarding the relationship between work disability and subsequent suicide or deliberate self-harm.

Review question

This scoping review aims to answer the following research question:

- What is the relationship between work disability and subsequent suicide or deliberate self-harm in working age people?

The review also seeks to answer a number of sub-questions, including:

- What personal, psychological, social, medical, environmental or other factors influence the relationship between work disability and subsequent suicide or deliberate self-harm?
- Is there evidence that certain approaches to the provision of social support for people with work disability are associated with subsequent suicide or deliberate self-harm?

Keywords

Work injury; Intentional self-harm; Workers' compensation; Mental health; Social security

Eligibility criteria

Participants

People aged 16 years or older with an episode or episodes of work disability.

Concepts

The core concept to be explored in this review are suicide and self-harm in people with work disability. Work disability is defined as a complete or partial incapacity to work due to an injury, illness or medical condition that can be temporary or permanent, and resulting in receipt in receipt of a financial payment from a third party such as an employer, insurance provider or government agency for a period of absence from work (e.g., a workers' compensation benefit, disability benefit, sickness absence payment, sick leave). Injury, illness, medical conditions that affect work capacity but do not result in a period of work absence will be excluded. Suicide, attempted suicide and self-harm are defined as taking or attempting to take one's own life or intentionally harming oneself. Death, injury or harm that arises from the acts of others, by illness or by accidental causes will be excluded.

Context

All settings and geographic locations will be considered for inclusion.

Types of Sources

This scoping review will consider both experimental and quasi-experimental study designs including randomized controlled trials, non-randomized controlled trials, before and after studies and interrupted time-series studies. In addition, analytical observational studies including prospective and retrospective cohort studies, case-control studies and analytical cross-sectional studies will be considered for inclusion.

Qualitative studies will also be considered that focus on qualitative data including, but not limited to, designs such as phenomenology, grounded theory, ethnography, qualitative description, action research and feminist research.

Methods

The proposed scoping review will be conducted in accordance with the method outlined by Arskey & O'Malley (14) and extended by Levac et al (15).

Search strategy

The search strategy will aim to locate published studies. An initial limited search of PubMed was undertaken to identify articles on the topic. The text words contained in the titles and abstracts of relevant articles, and the index terms used to describe the articles were used to develop a full search strategy (see Appendix 1). Search terms will be kept broad with limited exclusions to ensure all relevant literature is covered. The search strategy, including all identified keywords and index terms, will be adapted for each included database and/or information source. Forward citation searches of included studies will be conducted using academic databases. Backward citation searches from reference lists of included studies will also be conducted.

Studies published in English language will be included. Studies published since 1 January 2000 date will be included. The databases to be searched include Medline, PubMed and Scopus. Authors of included studies will be contacted to seek further studies for screening.

Source of Evidence selection

Following the search, all identified citations will be collated and uploaded into EndNote version 9.3 (Clarivate Analytics, PA, USA) and duplicates removed.

Following a pilot test, titles and abstracts will then be screened by two independent reviewers for assessment against the inclusion criteria for the review. Potentially relevant sources will be retrieved in full. The full text of selected citations will be assessed in detail against the inclusion criteria by two independent reviewers. Any disagreements that arise between the reviewers at each stage of the selection process will be resolved through discussion, or where agreement cannot be reached through discussion, will be adjudicated by an additional reviewer.

Reasons for exclusion of sources of evidence at full text that do not meet the inclusion criteria will be recorded and reported in the scoping review. The results of the search and the

study inclusion process will be reported in full in the final scoping review and presented in a Preferred Reporting Items for Systematic Reviews and Meta-analyses extension for scoping review (PRISMA-ScR) flow diagram (16).

Data Extraction

Data will be extracted from papers included in the scoping review using a data extraction tool developed by the reviewers. The data extracted will include specific details about the participants, concept, context, study methods and key findings relevant to the review question/s. A preliminary set of data fields is provided (see Appendix B). The draft data extraction tool will be modified and revised as necessary during the process of extracting data from each included evidence source. Modifications will be detailed in the scoping review.

Data extraction will initially be completed on five included studies by two reviewers. Extracted data will be reviewed by both reviewers to confirm that all relevant information is included and required to answer the research questions. Any disagreements that arise between the reviewers will be resolved through discussion, or with an additional reviewer/s. Once an approach to extraction is agreed upon, a single author will extract data for the remainder of included studies. If appropriate, authors of papers will be contacted to request missing or additional data, where required.

Data Analysis and Presentation

A descriptive numerical summary of the included studies will be developed and presented in a tabular format. This will describe the numbers of studies by key study features including by study design, country of origin, nature of condition leading to work disability, type of disability benefit etc..

A qualitative thematic analysis will then be undertaken using the authors conclusions and the results of any statistical analysis, regarding the relationship between work disability and suicide, and any factors that contribute to or modify this relationship.

We envisage that data will be reported in two sections. The first section will address the primary study question of the relationship between work disability and subsequent suicide and self-harm. The second section will address the review sub-questions.

For each of these sections a narrative summary will be developed, describing the main findings of the included studies and where possible, grouping studies according to the study outcome (suicide, self-harm) and/or by the nature of work disability (short-term, long-term), type of disability benefit scheme (cause-based, disability-based), or other features of the included studies. Where feasible a graphical summary of results will be developed. The narrative summary will accompany the tabulated and/or charted results and will describe how the results relate to the reviews objective and sub-questions.

Gaps in the current evidence base will also be identified and described in order to identify opportunities for future research.

Funding

The first author is supported by a Future Fellowship from the Australian Research Council (FT190100218). The funder played no role in the review process.

Conflicts of interest

The authors declare no conflicts of interest.

References

1. Collie A, Di Donato M, Iles R. Work Disability in Australia: An Overview of Prevalence, Expenditure, Support Systems and Services. J Occup Rehabil. 2018.

2. Paul KI, Moser K. Unemployment impairs mental health: Meta-analyses. Journal of Vocational Behavior. 2009;74(3):268-82.

3. Milner A, Page A, LaMontagne AD. Long-term unemployment and suicide: a systematic review and meta-analysis. PLoS One. 2013;8(1):e51333.

4. Collie A, Sheehan L, Lane TJ, Iles R. Psychological Distress in Workers' Compensation Claimants: Prevalence, Predictors and Mental Health Service Use. J Occup Rehabil. 2020;30(2):194-202.

5. Department of Social Services. DSS Statistical Summary (June 2018). In: Services DoS, editor. Canberra, Australia: Department of Social Services; 2018.

6. Grant GM, O'Donnell ML, Spittal MJ, Creamer M, Studdert DM. Relationship between stressfulness of claiming for injury compensation and long-term recovery: a prospective cohort study. JAMA Psychiatry. 2014;71(4):446-53.

7. Kilgour E, Kosny A, McKenzie D, Collie A. Interactions between injured workers and insurers in workers' compensation systems: a systematic review of qualitative research literature. J Occup Rehabil. 2015;25(1):160-81.

8. Lippel K. Workers describe the effect of the workers' compensation process on their health: A Quebec study. Int J Law Psychiat. 2007;30(4-5):427-43.

9. Sheehan LR, Lane TJ, Collie A. The Impact of Income Sources on Financial Stress in Workers' Compensation Claimants. J Occup Rehabil. 2020.

10. Kosny A, Newnam S, Collie A. Family matters: compensable injury and the effect on family. Disabil Rehabil. 2018;40(8):935-44.

11. Otsuka T, Tomata Y, Zhang S, Tanji F, Sugawara Y, Tsuji I. The association between emotional and instrumental social support and risk of suicide death: A population-based cohort study. J Psychiatr Res. 2019;114:141-6.

12. Collie A, Sheehan LR, McAllister A. Health service use of Australian unemployment and disability benefit recipients: a national, cross-sectional study. BMC Health Serv Res 21, 249 (2021). https://doi.org/10.1186/s12913-021-06255-0

13. Collie A, Newnam S, Keleher H, et al. Recovery within injury compensation schemes: A systems mapping study. J Occup Rehabil 2019 Mar;29(1):52-63.

14. Arksey H, O'Malley L. Scoping studies: towards a methodological framework. International Journal of Social Research Methodology. 2005;8:19-32.

15. Levac D, Colquhoun H, O'Brien KK. Scoping studies: advancing the methodology. Implement Sci. 2010;5:69.

16. Tricco A, Lillie A, Zarin W, et al. PRISMA Extension for Scoping Reviews (PRISMA-ScR): Checklist and Explanation. Ann Intern Med 2018; 169: 467-473.

Appendices

Appendix I: Search conducted 27 April 2021

Search number	Query	Records Retrieved
1	"work disability"[All Fields] OR "disability pension"[All Fields] OR "sickness absence"[All Fields] OR "sick leave"[All Fields] OR "workers compensation"[All Fields] OR "workman's compensation"[All Fields] OR "social assistance"[All Fields] OR "disability insurance"[All Fields] OR "social securit*"[All Fields] OR "wage replacement"[All Fields] OR "income benefit"[All Fields] OR "sick-listed"[All Fields] OR "short-term disability"[All Fields] OR "long-term disability"[All Fields]	41,474
2	"suicid*"[All Fields] OR "self injur*"[All Fields] OR ("self injurious behavior"[MeSH Terms] OR ("self injurious"[All Fields] AND "behavior"[All Fields]) OR "self injurious behavior"[All Fields] OR ("deliberate"[All Fields] AND "self"[All Fields] AND "harm"[All Fields]) OR "deliberate self harm"[All Fields])	109,902
3	#1 AND #2	301

Appendix II: Draft Data Extraction Fields

- Study Title
- Authors
- Journal
- Year of Publication
- Citation
- Country of origin
- Study aim / objective / purpose
- Study design (controlled trial, prospective cohort, retrospective cohort, cross-sectional, qualitative)
- Study inception period (date range)
- Study follow-up period
- Age range of sample
- Sex/Gender distribution of sample
- Sample size (N)
- Nature of injury/illness/condition leading to work disability (e.g., musculoskeletal disorder, traumatic injury, mental health condition)
- Work relatedness (i.e., whether work disability was acquired in the course of employment)

- Duration of work disability (temporary, permanent)
- Extent of work disability (full, partial)
- Type of work disability benefit (e.g., workers’ compensation, disability insurance, sick leave)
- Description of suicide and self-harm outcomes
    - Suicide, Attempted suicide, Deliberate self-harm
    - Prevalence / incidence of each outcome
    - Relative risk of each outcome
- List of covariates associated with suicide/self-harm and direction of effect
- Statistical / data analysis method
- Major themes (for qualitative studies)
- Author conclusions regarding relationship between work disability, suicide and self-harm.
- Implications for policy and practice
- Gaps identified by study authors
- Additional notes
