# Supplementary material for: The relationship between work disability and subsequent suicide or self-harm: A scoping review
Source: PLOS Glob Public Health. 2022 Dec 7;2(12):e0000922. doi: 10.1371/journal.pgph.0000922 (PMC10021753; doi:10.1371/journal.pgph.0000922)
Supplement: S3 Text — (DOCX) [file pgph.0000922.s003.docx]

# Appendices

### Appendix I: Search conducted 27 April 2021

| Search number | Query | Records Retrieved |
| --- | --- | --- |
| 1 | "work disability"[All Fields] OR "disability pension"[All Fields] OR "sickness absence"[All Fields] OR "sick leave"[All Fields] OR "workers compensation"[All Fields] OR "workman's compensation"[All Fields] OR "social assistance"[All Fields] OR "disability insurance"[All Fields] OR "social securit*"[All Fields] OR "wage replacement"[All Fields] OR "income benefit"[All Fields] OR "sick-listed"[All Fields] OR "short-term disability"[All Fields] OR "long-term disability"[All Fields] | 41,474 |
| 2 | "suicid*"[All Fields] OR "self injur*"[All Fields] OR ("self injurious behavior"[MeSH Terms] OR ("self injurious"[All Fields] AND "behavior"[All Fields]) OR "self injurious behavior"[All Fields] OR ("deliberate"[All Fields] AND "self"[All Fields] AND "harm"[All Fields]) OR "deliberate self harm"[All Fields]) | 109,902 |
| 3 | #1 AND #2 | 301 |

### Appendix II: Draft Data Extraction Fields

- Study Title
- Authors
- Journal
- Year of Publication
- Citation
- Country of origin
- Study aim / objective / purpose
- Study design (controlled trial, prospective cohort, retrospective cohort, cross-sectional, qualitative)
- Study inception period (date range)
- Study follow-up period
- Age range of sample
- Sex/Gender distribution of sample
- Sample size (N)
- Nature of injury/illness/condition leading to work disability (e.g., musculoskeletal disorder, traumatic injury, mental health condition)
- Work relatedness (i.e., whether work disability was acquired in the course of employment)
- Duration of work disability (temporary, permanent)
- Extent of work disability (full, partial)
- Type of work disability benefit (e.g., workers’ compensation, disability insurance, sick leave)
- Description of suicide and self-harm outcomes
  - - Suicide, Attempted suicide, Deliberate self-harm
    - Prevalence / incidence of each outcome
    - Relative risk of each outcome
- List of covariates associated with suicide/self-harm and direction of effect
- Statistical / data analysis method
- Major themes (for qualitative studies)
- Author conclusions regarding relationship between work disability, suicide and self-harm.
- Implications for policy and practice
- Gaps identified by study authors
- Additional notes
