# Supplementary material for: The relationship between work disability and subsequent suicide or self-harm: A scoping review
Source: PLOS Glob Public Health. 2022 Dec 7;2(12):e0000922. doi: 10.1371/journal.pgph.0000922 (PMC10021753; doi:10.1371/journal.pgph.0000922)
Supplement: Attachment — Submitted filename: response to reviewers.docx [file pgph.0000922.s004.docx]

The Editor

PLOS Global Public Health

via online submission

14^th^ July 2022

Dear Editor,

Re: “The relationship between work disability and subsequent suicide or self-harm: A systematic scoping review”

Thank-you for sourcing two constructive reviews of this manuscript. We thank the reviewers for taking the time to comment on the manuscript and for their valuable comments.

We also note the email correspondence with you (dated 13 May 2022) clarifying that you were not requesting that we revise the manuscript as a systematic review, but rather that we more systematically report the review methods, using the PRISMA guidelines.

We have revised the manuscript, taking into account the reviewer’s comments and your editorial guidance. Please find attached to this letter a point-by-point response to the reviewer’s comments. Our responses are in *italics* and indented.

We have uploaded the revision as per your instructions.

I hope you find the revision acceptable.

Sincerely,

Professor Alex Collie

Director, Healthy Working Lives Research Group

Australian Research Council Future Fellow

School of Public Health and Preventive Medicine

Monash University

Email - [alex.collie@monash.edu](mailto:alex.collie@monash.edu)

**RESPONSE TO REVIEWERS**

**Reviewer #1:**

The current scoping review has compiled the literature on the association between work disability and suicidal/ self-harm outcomes, and used a narrative analysis to explore the nature and outcome of those studies.

Title: Clear and adequate

Abstract: Under Methods, please mention the databases from where the data were obtained, along with other data sources.

*The abstract has been updated.*

Introduction: Adequate

Methods:
In the manuscript, individuals of 15 years and more with working disability are included, but in the proposal used for registration, the lower age limit was mentioned as 16. Is there any reason for change this criterion? If yes, please justify the reason for the choice of the age range.

*As noted by the reviewer this is a deviation from the study protocol We have explained in the revision that this occurred as we identified studies that otherwise met all inclusion criteria but that included participants as young as 15 years of age (page 7, line 159-161).*

If supplementary table I (page 8, line 171) is the Appendix I, please name it and place it accordingly.

*We have clarified that this is Supplementary File 2. As per journal requirements, this is provided as a separate file and not included in the body of the manuscript.*

Did the authors check the overlap of data obtained through three different methods – systematic search, reference chaining, and expert recommendations?

*Yes we did. There was relatively little overlap. Two of the included papers identified through expert review were also identified in the systematic search of databases. These were excluded when duplicates were removed. As described in the search strategy, reference chaining (forwards and backwards citation searching) occurred on those studies that met inclusion criteria. The purpose of this was to seek additional studies, beyond those already included. Thus by definition there is no overlap between studies identified using reference chaining and those identified using other methods.*

If the systematic search from major databases combined with a manual search would yield sufficient data for a systematic review, what was the purpose of requesting expert recommendations?

*As this is a scoping review seeking studies using a relatively diverse range of methods (quantitative, qualitative etc.) across multiple disciplinary areas, we sought to conduct a broad search to ensure we captured all relevant literature. We considered that seeking expert recommendations would complement the other methods and help to ensure we captured all relevant literature.*

*We have included an additional sentence at the beginning of the search strategy section to make this point more clearly.*

Considering that the study focuses on a specific research question, there was a possibility of systematic review, adopting PRISMA, that would have enhanced the robustness of the study and its outcome. So, why did the authors opt for scoping review? Please describe the reason in the Methods section with justification.

*Our justification for adopting a scoping review method is provided in the introduction section, lines 129-134, as follows: “While the existing evidence is highly suggestive of a link between work disability and later suicide and self-harm, the evidence-base is disaggregated. Studies have been conducted using a variety of methods, in multiple cultural and societal contexts, using a variety of key concept and outcome definitions, and across nations with very differing approaches to supporting people with work disability. There is a need for a scoping study to examine the relationship between work disability and subsequent suicide and self-harm.”*

*PRISMA is a reporting standard for systematic reviews, including systematic scoping reviews. It is not a method for conducting systematic reviews. We did adopt the PRISMA reporting approach and did submit our PRISMA-ScR checklist with the original manuscript, but this does not seem to have been provided to the journal during the initial review process. We have re-attached the PRISMA-ScR checklist to the revision, and include the following additional sentence at the beginning of the methods section “We report the scoping review using the Preferred Reporting Items for Systematic Reviews and Meta-Analysis extension for Scoping Reviews (PRISMA-ScR). A PRISMA-ScR checklist is provided in Supplementary File 2.”*

Results:
In page 14, under sample definition, age range from 15 is mentioned twice as ‘adults’. The same is repeated in Table 3, from the age of 16. Do the authors consider participants aged 15 or 16 as adults? In such contexts, it is better to replace the word ‘adults’ with ‘individuals’ or ‘participants’.

*We have updated the tables to replace the term ‘adults’ with ‘individuals’ where the included age range was less than 18 years.*

Did the authors call out DP and SA? Please include the full form of the acronyms at the first time of its appearance in the manuscript.

*The first appearance of these terms is in the methods section, under evidence synthesis. We have defined these acronyms now in this section.*

Discussion: Adequate

Conclusion: The major finding(s) of the current review other than the association between work disability and self-harm tendency should be highlighted, especially because it is already an established fact as the current study has also pointed out.

*We have revised the conclusion to include more information on the risk factors/predictors of suicidal behaviour in work disabled people, and also to note the evidence of administrative burden as a risk factor for later suicidal behaviour. Given the length of the paper, we were conscious of reducing the duplication/re-statement of information in the discussion section within the concluding paragraph, so have chosen to keep this section brief.*

Reviewer #2: Overview:
This is a useful scooping review which has some good implications for practice and research. However, there are some areas which I feel the authors could benefit from, which would drive home the systematic nature of the review.
Primarily these are streamlining arguments and critical discussion, reviewing their screening process to ensure missing papers are accounted for, adding additional details to the methodology, and finally adding critical appraisal to the review.

General comments:
- Variation in terms used; scooping systematic review, systematic review – need consistency

*We have reviewed the manuscript and reverted to the term scoping review throughout.*

Abstract:
- Authors could dedicate some space to the methods; which databases were used?

*Please see response to same point raised by Reviewer #1.*

- Add protocol OSF link to methods and PRISMA-ScR.

*This information has been added to the abstract.*

Introduction:
- The introduction could benefit from being more specific (e.g. what conditions are associated with work disability?

*We have added additional detail in the first paragraph of the introduction on this topic, and included additional citations.*

What are the studies which show substantial proportion of people have mental health conditions?)

*The sentence following this statement in the introduction cites three studies. We have modified the text slightly to more clearly indicate that this sentence follows on and expands on the preceding sentence.*

….and highlighting evidence to support their arguments more closely (e.g. line 113; “financial hardship is frequently cited as a risk factor for suicidal behaviour.” But there is no reference following this statement)

*We have included a citation following this statement.*

- Paragraph 3 which focuses on linking work disability and suicide/self-harm seems a bit unfocused. Authors have a really nice argument in paragraph 4 (lines 125 – 128), potentially if this started paragraph 3, it would more neatly set up why paragraph 3 seems draws on risk factors rather than focus suicide data.

*Thank you that is a helpful suggestion for re-organising the introduction and we have taken this approach in the revision.*

- Paragraph 4; authors may want to include a definition of self-harm (and suicide), and rationale as to why these are used.

*We have provided a definition of the outcomes including suicide and self-harm in the methods section (eligibility criteria).*

Methods:
- Double referencing on line 140

*This has been fixed.*

- Was non-suicidal self-injury included in self-harm? This is unclear with the current eligibility criteria.

*We have added more detail to indicate that self-injury resulting in hospitalisation was also included. In practice we found it was not often possible to determine if there was suicidal intent in those studies that reported self-harm outcomes, so the operational definition we used was self-injury resulting in hospitalisation or suicide attempts. This has also been noted as a limitation in the discussion.*

- Abstract mentions workers above 15, was this a selection criterion or a finding?

*Please see response to question on age range from Reviewer #1.*

- Selection of sources of evidence: add numbers of paper selection at each stage. What was the percentage of author agreement between papers?

*The numbers for each stage of the selection process is reported in the results, in the first paragraph. We have added further information on author agreement to this section.*

- Why wasn’t there any critical appraisal of individual sources of evidence? This is asked for in the PRISMA-ScR and standard across reviews. Would recommend inclusion of this.

*We note that a critical appraisal is optional in a scoping review, and as per Munn et al (2018) is generally not performed. It is true that the PRISMA-ScR checklists requests authors to indicate if critical appraisal was conducted, but also to justify it and explain how the information was used in evidence synthesis. We included a wide variety of study designs which would have required use of a number of different appraisal tools, and this would have added complexity to an already complex evidence synthesis. Given that we did not have a specific objective of assessing evidence quality, we chose not to conduct critical appraisal.*

*Munn, Z., Peters, M.D.J., Stern, C. et al. Systematic review or scoping review? Guidance for authors when choosing between a systematic or scoping review approach. BMC Med Res Methodol 18, 143 (2018). https://doi.org/10.1186/s12874-018-0611-x*

- I would expect PRISMA flow chart in the methods, as well as paragraph 1 from Results.

*Figure 1 (the PRISMA flowchart) is first referenced in the methods, under the ‘search strategy’ heading. We have included a recommendation to the journal to insert the figure at this point.*

- PRISMA flowchart 524-457 = 67, chart shows 65 appear to have lost 2 papers.

*Thank you for picking this up. We re-checked our records and 459 were excluded at initial screening. We have updated the flowchart and manuscript accordingly.*

- What was the analysis method for the narrative review?

*Our approach to synthesis and data analysis is described in the final two paragraphs of the methods section. Essentially, we first constructed an evidence map, counting included studies. This led to the decision to focus synthesis by system of income support (WC, DP, SA). Then within each system we constructed a description of the included evidence, describing the relationship between work disability and suicide (study main objective), and any factors that contribute to or modify this relationship (key research question). Please refer to the final two paragraphs of the methods for more information – we have added a little more detail to make the approach clearer.*

- Is extracted data fully available? Tell us in the methods!

*We have uploaded extracted data to the Open Science Foundation project page (a public repository) and included a note with the link in the methods.*

- The methods feels a bit thin – I’d like to see rationale as to why authors made specific decisions.

*We have included some additional detail in response to prior comments, and we note that the review protocol is publicly available, and we have explained the major decisions that were not foreshadowed in the protocol (such as why we chose to synthesise evidence by system of income support). It is otherwise difficult to respond to this comment without specific suggestions from the reviewer.*

Results:
- A large section of results is descriptive of the papers, a lot of this is summarised in tables. Authors could streamline this to enhance engagement of the reader.

*We have made attempts to streamline the results section, by removing some text that repeats information presented in the tables.*

- Results would benefit from critical review of the data instead of simply listing individual study findings.

*The included literature is very diverse, in methods, settings and populations included. We do not simply list individual studies. We report results in groups, as per the narrative synthesis method. We discuss individual studies where this is appropriate or where studies do not logically fall into a grouping.*

*We refer the reviewer to the discussion section of the manuscript, which contains a critical review of the study findings. We think is the most appropriate place for such a review, and that the results section should instead present study findings in a way that is consistent with aims, in this case to identify the types of available evidence, examine how research is conducted, identify knowledge gaps and to generally develop a more fulsome understanding of this evidence base (as per the objectives of a scoping review).*

Discussion:
- No critical appraisal was discussed but then quality issue is mentioned in the discussion. To be systematic this should be done with all papers with clear indication of results. The appraisal tool should be available as a supplementary material so that readers can see the criteria for high-mod-low quality. This would support reproducibility of this paper as well.

*As noted above critical appraisal was not conducted. We have clarified this in the discussion and made it clearer that we are reporting general observations on study methods rather than the results of critical appraisal.*

- Was there any difference between self-harm and suicide related results? Any differences between behaviour and ideation.

*We have noted the relative lack of studies on self-harm and on ideation as an important evidence gap requiring further study. Please refer to the second last paragraph of the discussion “The evidence base is larger when suicide is the measured outcome, than for self-harm as an outcome, where there are fewer studies. We identified even fewer studies reporting suicidal ideation in work disabled samples. There is value in filling these evidence gaps in order to better understand the relationship between work disability and suicide or self-harm within work disability systems and within nations. Further studies on suicidal ideation would provide critical information to explore the pathway from work disability through suicidal ideation to completed suicide, which may inform preventive interventions.”*

- Overall, arguments are clear. I think again, authors are a bit descriptive of the papers results, rather than critically discussing them and how they draw together to form implications. However, there is some clear implications from this study which is great. Pinpointing the these are very worthwhile.

*Thank-you. We consider that the discussion reflects the purpose and use of a scoping review method, e.g., to identify the types of available evidence, examine how research is conducted, identify knowledge gaps. We have made efforts to refine the discussion, but have been cautious with our statements re implications given that this is not a systematic review.*
